# Opioid use by cancer status and time since diagnosis among older adults enrolled in the Prostate, Lung, Colorectal, and Ovarian screening trial in the United States

**DOI:** 10.1002/cam4.3810

**Published:** 2021-02-26

**Authors:** Danielle D. Durham, Scott A. Strassels, Paul F. Pinsky

**Affiliations:** ^1^ Department of Radiology University of North Carolina at Chapel Hill Chapel Hill NC USA; ^2^ Cancer Prevention Fellowship Program Division of Cancer Prevention Healthcare Delivery Research Program Division of Cancer Control and Population Sciences National Cancer Institute Bethesda MD USA; ^3^ Center for Surgical Health Assessment Research and Policy Department of Surgery The Ohio State University Columbus OH USA; ^4^ Division of Cancer Prevention National Cancer Institute Bethesda MD USA

**Keywords:** cancer, older adults, opioids, PLCO

## Abstract

**Background:**

Dosing limits in opioid clinical practice guidelines in the United States are likely misapplied to cancer patients, however, opioid use may be difficult to ascertain as they are largely excluded from opioid use studies.

**Methods:**

The primary objective was to determine whether cancer patients were more likely to be chronic opioid users after diagnosis. We described prescription opioid use among U.S. older adult cancer patients during two time periods, within 2 years of diagnosis (short‐term) and at least 2 years beyond diagnosis (long‐term), compared to those without cancer (controls). Among participants in the Prostate, Lung, Colorectal, and Ovarian (PLCO) screening trial with linkages to Medicare Part D data during 2011–2015, we used multivariable logistic regression to estimate the association between cancer diagnosis and opioid use outcomes controlling for demographics. The primary outcome of opioid use was measured with the following metrics: Any opioid use, chronic use (90 consecutive days supply of opioid use while allowing for a 7‐day gap between refills), high use (average daily morphine equivalent (MME) ≥120 mg for any 90‐day period), and total MME dose above 2,000 mg (MME_2000_).

**Results:**

The short‐term cohort included 1,491 cancer patients and 24,930 controls. Any use in the 2‐year post‐diagnosis period was higher among cancer patients OR 3.3 (95% CI: 3.0–3.7). Chronic use rates were similar by cancer status (4.6% vs. 3.8% for cases and controls, respectively). The long‐term cohort included 4,377 cancer patients and 27,545 controls. Rates of any use were similar among cancer patients and controls (63% vs. 59%).

**Conclusions:**

Any opioid use was similar among long‐term cancer survivors compared to controls, but differed among short‐term survivors for any opioid use and marginally for chronic opioid use.

## Graphical abstract

Use of any opioid was similar among long‐term cancer survivors compared to controls, but differed among short‐term survivors for any opioid use and marginally for chronic opioid use. Continued monitoring of opioid use among subgroups of cancer patients is important. This should include concomitant use of opioids and non‐opioid medications commonly used to manage pain among cancer patients.

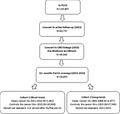

## 1 BACKGROUND

Pain is an anticipated consequence of cancer treatment and disease progression. Approximately 38% of cancer patients report moderate to severe pain^1^ with variations by pain type and disease characteristics.^2^, ^3^ Pain management via opioids is important for cancer patients who have severe pain not adequately managed by alternative medications. Cancer patients may have varying patterns of pain management when compared to patients without cancer.^4^

Debate regarding opioid use continues due to concerns for the balance between appropriate use, adverse effects of chronic use, and risk of misue.^5^, ^6^ A potential unintended consequence is that patients who benefit from pain management via properly prescribed and used opioids may not receive it as a result of tightened prescribing recommendations generally^7^, ^8^, ^9^ and for acute pain.^10^, ^11^

Development of persistent use after cancer treatment among opioid‐naïve patients is another concern.^12^, ^13^, ^14^, ^15^ Pain management is integral to cancer care,^16^, ^17^ however, opioid dose limits in clinical practice guidelines for non‐cancer pain have been misapplied to cancer and palliative care patients.^7^, ^8^, ^9^ Efforts to manage opioid misuse may have unintended consequences including complex pain and inadequate pain relief through diagnosis, treatment, and survivorship.^18^, ^19^, ^20^ Possibly illustrating this point, a Canadian study found no change in opioid prescribing over time among older cancer patients while opioid prescribing increased among non‐cancer patients.^21^ A study of outpatient oncology patients found 33% of patients with pain reported receiving inadequate pain management prescribing.^22^ Prescription regulations and policies may impact medication prescribed to cancer patients.^23^ Inadequate pain management may have negative consequences on health outcomes.^24^, ^25^

Cancer patients are often excluded from opioid use studies.^26^ Describing pain management may provide insights as to how opioids are used in cancer care and may identify points in the cancer care continuum to ensure appropriate pain management. We examined prescription opioid use among cancer patients at two time points around cancer diagnosis compared to those without cancer to determine whether cancer patients were more likely to be chronic opioid users after diagnosis.

## 2 METHODS

### 2.1 Data sources and study population

This is a secondary analysis from the Prostate, Lung, Colorectal, and Ovarian (PLCO) Cancer Screening Trial.^27^ Participants were enrolled at 10 U.S. screening centers from 1993 to 2001. Eligible study participants were between 55 and 74 years of age with no previous diagnosis of trial cancers. Participants provided informed consent and each screening center's Institutional Review Board approved the trial. Demographic information was available from a baseline and supplemental questionnaire completed in 2006–2007.

Participants were followed up to 13 years for all‐cancer incidence and mortality. In 2011, PLCO transitioned to centralized follow‐up. Participants reconsented to continue active follow‐up, switch to passive follow‐up, or refused further follow‐up. The active follow‐up group was recontacted with questionnaires or consent requests. In 2013, the active group was asked to consent for linkage to Medicare claims. Active and passive participants continued to be followed for mortality and cancer incidence through 2015 based on linkage with the National Death Index and U.S. state cancer registries.

### 2.2 Part D Medicare claims data

Information on prescription medication use was derived from linked Medicare Part D claims for the years 2011–2015. Part D contains information for each filled prescription including quantity, days’ supply, and drug strength. For each linked PLCO study participant, we calculated the morphine milligram equivalents (MME) for each fill and summed over all prescriptions to obtain MME dosage over the relevant study period using CDC conversion tables for analyzing prescription data.^28^ Prescription opioid use outcomes of interest were: any opioid use, chronic opioid use, high opioid use, and total MME dose above 2,000 mg (MME_2000_). Any opioid use was defined as having at least one claim for an opioid prescription fill during the study window. Chronic opioid use was defined as having a record of 90 consecutive days’ opioid supply while allowing for a 7‐day gap between refills.^29^ High opioid use was defined as an average daily MME of at least 120 mg over any consecutive 90‐day period and at least 90 days of opioid use during the time period of interest.^30^, ^31^

### 2.3 Statistical analysis

We created two study cohorts to assess opioid use. Participants were excluded if they had <12 months Part D coverage during the assessment periods to ensure sufficient time to assess opioid use. To compare opioid use among short‐term cancer survivors, we selected those with any cancer diagnosis between 1 January 2011 and 31 December 2013 (ensuring the potential for at least 2 years of post‐diagnosis follow‐up to assess Medicare Part D claims), and those without a cancer diagnosis through the end of 2015 as controls (Cohort 1). For cancer patients, opioid use was assessed for the 2‐year period following diagnosis, while for controls use was assessed for the 2‐year period from their pseudo‐diagnosis date (i.e., a randomly selected date between 1 January 2011 and 31 December 2013). The demographic distribution of cancer patients included in the current study were generally representative of all potentially eligible cases (Tables A2 and A3). To determine whether cancer patients were more likely than controls to have had preexisting conditions predisposing them to opioid use, we assessed use in the year before diagnosis or pseudo‐diagnosis. We examined the period 6–18 months prior to diagnosis, since cancer symptoms could have been present before diagnosis.

To compare opioid use among long‐term cancer survivors (diagnosis more than 2 years before the start of the assessment period) to controls, we selected those with a cancer diagnosis from randomization date until 31 December 2008, and those with no cancer diagnosis through 31 December 2015 as controls (Cohort 2). Opioid use was assessed for the period 2011–2015.

To estimate the association between cancer diagnosis and the opioid use outcomes we used multivariate logistic regression to calculate odds ratios (ORs). The base model controlled for age and sex; the adjusted model additionally controlled for education, race/ethnicity, income, and marital status.

Because the distribution of cancer types differed by sex, analyses were stratified by sex. The statistical significance of interactions of cancer status by sex were assessed. Since prostate and breast cancer constituted large proportions of the cancers in men and women, respectively, sex‐specific analyses were performed stratified by cancer type (breast or prostate vs. all other). Within sex, statistical tests were performed for whether ORs differed by cancer type.

Analyses were conducted using SAS software version 9.4. SAS Institute Inc., Cary, NC.

## 3 RESULTS

Of the 154,887 participants enrolled in PLCO, 49,560 consented to Medicare linkage and 35,855 had at least 12 months of Medicare Part D coverage (Figure A1). Cohort 1 included 1,491 short‐term cancer survivors (first cancer diagnosis from 1 January 2011 through 31 December 2013) and 24,930 controls. Cohort 2 included 4,377 long‐term cancer survivors (diagnosis from randomization until 31 December 2008) and 27,545 controls. In Cohort 1, cancer patients were more likely than controls to be men (53.8% vs. 42.0%), and cancer patients were slightly older. In Cohort 2, cancer patients were slightly older than controls (median ages 75 and 73, respectively) and substantially more likely to be men (60.9% vs. 42.5%; Table 1).

**TABLE 1:** Demographic distribution of study population by cancer status and time since cancer diagnosis (Cohort 1 & 2)

		Cohort 1		Cohort 2	
		Short‐term cancer survivors N = 1,491	Short‐term controls N = 24,930	Long‐term cancer survivors N = 4,377	Long‐term controls N = 27,545
		N (col %)	N (col %)	N (col %)	N (col %)
Age at start of analysis period	Median (IQR)	75 (71/80)	75 (71/79)	75 (71/79)	73 (69/78)
Sex	Men	802 (53.8)	10,469 (42.0)	2,666 (60.9)	11,714 (42.5)
	Women	689 (46.2)	14,461 (58.0)	1,711 (39.1)	15,831 (57.5)
Education	Less than high school	48 (3.2)	887 (3.6)	147 (3.4)	973 (3.5)
	High school graduate	258 (17.3)	4,872 (19.5)	752 (17.2)	5,385 (19.6)
	Post high school	497 (33.3)	8,085 (32.4)	1,372 (31.4)	8,918 (32.4)
	College graduate	665 (44.6)	10,640 (42.7)	2,041 (46.6)	11,775 (42.8)
	Unknown	23 (1.5)	446 (1.8)	65 (1.5)	494 (1.8)
Race/ethnicity	White, non‐Hispanic	1,353 (90.7)	22,216 (89.1)	3,946 (90.2)	24,547 (89.1)
	Black, non‐Hispanic	28 (1.9)	538 (2.2)	114 (2.6)	654 (2.4)
	Hispanic	17 (1.1)	351 (1.4)	52 (1.2)	390 (1.4)
	Asian	60 (4.0)	1,227 (4.9)	177 (4.0)	1,299 (4.7)
	Pacific Islander/American Indian	12 (0.8)	190 (0.7)	32 (0.7)	204 (0.7)
	Unknown	21 (1.4)	408 (1.6)	56 (1.3)	451 (1.6)
Income	<$20,000	89 (6.0)	2,079 (8.3)	274 (6.3)	2,224 (8.1)
	$20,000–$49,000	484 (32.5)	8,039 (32.3)	1,344 (30.7)	8,846 (32.1)
	$50,000–$99,000	443 (29.7)	6,623 (26.6)	1,271 (2.0)	7,392 (26.8)
	>$100,000	153 (10.3)	2,255 (9.1)	449 (10.3)	2,515 (9.1)
	Unknown	322 (21.6)	5,934 (23.8)	1,039 (23.7)	6,568 (23.8)
Marital status	Married/living as Married	1,103 (74.0)	18,021 (72.3)	3,377 (77.2)	19,996 (72.6)
	Widowed	172 (11.5)	3,173 (12.7)	495 (11.3)	3,457 (12.6)
	Divorced/separated	151 (10.1)	2,852 (11.4)	370 (8.5)	3,124 (11.3)
	Never married	68 (4.6)	796 (3.2)	119 (2.7)	870 (3.2)
	Unknown	5 (0.3)	88 (0.4)	16 (0.4)	98 (0.4)
Comorbidities^a^	0	571 (38.3)	9,370 (37.6)	1,565 (35.8)	10,400 (37.8)
	1	577 (38.7)	10,120 (40.6)	1,787 (40.8)	11,113 (40.3)
	2	185 (12.4)	2,866 (11.5)	523 (11.9)	3,148 (11.4)
	Unknown	158 (10.6)	2,573 (10.3)	502 (11.5)	2,884 (10.5)

^a^ Number of comorbidities. Includes myocardial infarction (MI), stroke, diabetes, emphysema, arthritis, broken hip/vertebra.

### 3.1 Cohort 1 results

For Cohort 1, mean (SD) months of part D coverage during the assessment period was 23.0 (2.7) and 23.4 (2.2) for cancer patients and controls, respectively. Among cancer patients, for men, 40% were prostate cancer and for women 36% were breast cancer (Table A1).

Rates of any use were higher for cancer patients (68.4%) than for controls (40.2%), as were MME_2000_ rates (14.6% vs. 7.9%). In contrast, rates of chronic opioid use were similar by cancer status (4.6% vs. 3.8% for cases and controls, respectively). High opioid use was infrequent in both groups but significantly higher among cancer patients (1.1% vs. 0.3%). For both cancer patients and controls, rates of any use, chronic use, and MME_2000_ were higher in women than men, but with similar distribution among cancer patients compared to controls (Table 2). Median MME dose among users was similar for cases and controls. The most commonly prescribed opioid medications for cancer patients were products containing hydrocodone or oxycodone, and tramadol (Tables A4 and A5). Compared to controls, cancer patients had a higher proportion of oxycodone prescriptions and a lower proportion of tramadol prescriptions (Table A2).

**TABLE 2:** Opioid prescription use by cancer status for Cohort 1; Use in 2‐year period for short‐term cancer survivors versus controls without a cancer diagnosis

	All		Men		Women	
	Cancer diagnosis 2011–2013	No Cancer diagnosis through 2015	Cancer diagnosis 2011–2013	No Cancer diagnosis through 2015	Cancer diagnosis 2011–2013	No Cancer diagnosis through 2015
Total in Group	1,491	24,930	802	10,469	689	14,461
≥1 opioid fill (any use during study period), N (%)	1,020 (68.4)	10,025 (40.2)	506 (63.1)	3,925 (37.5)	514 (74.6)	6,100 (42.2)
Median MME dose in mg among Users (IQR)	378 (178/1,480)	375 (150/1,340)	390 (200/1,350)	300 (150/1,000)	375 (150/1,510)	450 (150/1,645)
Chronic use^a^, N (%)	68 (4.6)	941 (3.8)	26 (3.2)	284 (2.7)	42 (6.1)	657 (4.5)
High use^b^, N (%)	16 (1.1)	73 (0.3)	8 (1.0)	28 (0.3)	8 (1.2)	45 (0.3)
MME_2000_ ^c^, N (%)	218 (14.6)	1,972 (7.9)	102 (12.7)	616 (5.9)	116 (16.8)	1,356 (9.4)

Abbreviation: MME, morphine milligram equivalent.

^a^ Chronic opioid use defined as 90 consecutive days of opioid use allowing got a 7‐day gap between refills.

^b^ High opioid use defined as an average daily MME dose ≥120 mg for any 90‐day period.

^c^ Total MME dose >2,000 mg.

Among men with cancer, MME_2000_ significantly differed between prostate and non‐prostate cancer patients, with rates of 6.6% versus 16.8%, respectively (*p* < 0.0001). Among women, for breast cancer compared to non‐breast cancer patients, the former had significantly lower rates of chronic use (3.2% vs. 7.7%, *p* = 0.02), high use (0% vs. 1.8%, *p* = 0.03), and MME_2000_ (8.4% vs. 21.6%, *p* < 0.0001).

Within the 2‐year window, any use rates for cases were 59.8% in the first year and 32.0% in the second year; comparable rates for controls were 26.6% and 27.7%. In the period 6–18 months prior to diagnosis or pseudo‐diagnosis, opioid use was assessed among the 82% of cases and 81% of controls with complete part D coverage during the period. Opioid use was similar in each group; rates of any use were 26.7% for cases versus 26.6% for controls, and rates of MME_2000_ were 4.6% for cases versus 4.7% for controls.

Odds ratios were similar for the base and adjusted models. The OR for any opioid use was elevated, with an OR of 3.3 (95% CI: 3.0–3.7); by sex, the OR was significantly higher for women (4.0) than men (2.8) (*p*‐value for interaction = 0.003). ORs were significantly elevated for MME_2000_, with ORs slightly higher among men when compared to women (adjusted OR 2.3 vs. 2.0, respectively). For chronic use, the OR was modestly elevated (OR = 1.32; 95% CI: 1.02–1.70; Table 3).

**TABLE 3:** Odds ratio (OR) estimates for opioid use for short‐term cancer survivors versus controls without a cancer diagnosis in Cohort 1

	All		Men		Women		*p*‐value interaction (sex by cancer status)
	Base^a^	Adjusted^b^	Base^a^	Adjusted^b^	Base^a^	Adjusted^b^	Adjusted^b^
	OR (95% CI)						
Any opioid use	3.3 (3.0–3.7)	3.3 (3.0–3.7)	2.8 (2.5–3.3)	2.8 (2.4–3.3)	4.0 (3.4–4.8)	4.1 (3.4–4.9)	0.002
Chronic use^c^	1.30 (1.01–1.67)	1.32 (1.02–1.70)	1.20 (0.8–1.8)	1.18 (0.8–1.8)	1.36 (0.99–1.9)	1.40 (1.02–1.9)	0.57
High use^d^	3.8 (2.2–6.6)	3.8 (2.2–6.7)	3.8 (1.7–8.5)	3.8 (1.7–8.4)	3.8 (1.8–8.1)	3.8 (1.8–8.1)	0.98
MME_2000_ ^e^	2.1 (1.8–2.5)	2.1 (1.8–2.5)	2.3 (1.9–2.9)	2.3 (1.8–2.9)	1.9 (1.6–2.4)	2.0 (1.6–2.5)	0.35

Note: Referent category is individuals without a cancer diagnosis during the study window.

Abbreviation: MME, morphine milligram equivalent.

^a^ Model adjusted for sex (in non‐sex‐specific analyses) and age.

^b^ Model additionally adjusted for education, race/ethnicity, income, and marital status.

^c^ Chronic opioid use defined as 90 consecutive days of opioid use allowing for 7‐day gap between refills.

^d^ High use defined as greater than or equal to 120 mg or any 90‐day period and at least 90 days of opioid use.

^e^ Total MME dose >2,000 mg.

Within men, the OR differed significantly by cancer type (prostate vs. non‐prostate) for MME_2000_, with ORs of 1.1 (prostate) versus 3.2 (non‐prostate; *p* < 0.0001). For women, the ORs differed significantly by cancer type (breast vs. non‐breast) for chronic use (ORs = 0.7 and 1.8, respectively; *p* = 0.03) and for MME_2000_ (ORs = 0.9 and 2.7, respectively, *p* < 0.0001).

### 3.2 Cohort 2 results

For Cohort 2, the mean (SD) length of part D coverage was 52.7 (13.2) and 53.2 (12.7) months for cancer patients and controls, respectively. The median (25^th^/75^th^) time from cancer diagnosis to the start of the assessment period for cases was 7.0 (4.3/10.1) years. In men, 67% of diagnoses were prostate cancer while in women 48% were breast cancer (Table A1). Similar to the short‐term cohort, the most common opioid medications prescribed were tramadol and products containing hydrocodone or oxycodone. The distribution of medications was similar for cases and controls. (Tables A4 and A5).

Rates of any opioid use were 63.1% for cancer patients versus 59.0% for controls. Use rates were higher for women than men among both cases and controls, and for each sex, slightly higher for cancer cases than controls (Table 4). Chronic opioid use was similar by cancer status; 5.2% versus 5.4% overall for cases and controls, respectively; chronic use was higher in women for both cases and controls. High opioid use was rare (<1% for both cases and controls).

**TABLE 4:** Opioid prescription use by cancer status for Cohort 2; Use in 5‐year period for long‐term cancer survivors versus controls without a cancer diagnosis

	All		Men		Women	
	Cancer diagnosis through 2008	No Cancer diagnosis through 2015	Cancer	No Cancer	Cancer	No Cancer
Total in group	4,377	27,545	2,666	11,714	1,711	15,831
≥1 Opioid fill (any use during study period), N (%)	2,760 (63.1)	16,242 (59.0)	1,633 (61.3)	6,616 (56.5)	1,127 (65.9)	9,626 (60.8)
Median MME dose in mg among users (IQR)	500 (200/1,540)	495 (197/1,625)	450 (180/1,280)	400 (150/1,200)	592 (225/2,050)	596 (200/1,980)
Chronic use^a^, N (%)	226 (5.2)	1,484 (5.4)	111 (4.2)	447 (3.8)	115 (6.7)	1,037 (6.6)
High use^b^, N (%)	23 (0.53)	121 (0.44)	9 (0.3)	42 (0.4)	14 (0.8)	79 (0.5)
MME_2000_ ^c^, N (%)	594 (13.6)	3,536 (12.8)	308 (11.6)	1,137 (9.7)	286 (167)	2,399 (15.2)

Note: “All use” is for 2011–2015.

Abbreviation: MME, morphine milligram equivalent.

^a^ Chronic opioid use defined as 90 consecutive days of opioid use allowing got a 7‐day gap between refills.

^b^ High use defined as average daily MME dose ≥120 mg for any 90‐day period.

^c^ Total MME dose >2000 mg.

No opioid use metric differed significantly between breast and non‐breast cancer cases for women or between prostate and non‐prostate cancer cases for men. Annual rates of any use varied little over the 2011–2015 period, with a range of annual rates of 26.3–30.3 for cases and 26.2–27.6 for controls. For cases, rates of any use in the period varied little by time since cancer diagnosis (range 61–65% by quartile of time since diagnosis).

Odds ratios were similar for the base and adjusted model. In the adjusted model, for men and women combined, the OR for any use was significantly elevated, though of modest magnitude (OR = 1.23, 95% CI: 1.15–1.32), as was the OR for MME_2000_ (OR = 1.17, 95% CI: 1.06–1.29; Table 5). ORs were not significant for chronic or high use. There were no significant interactions of sex by cancer status. For MME_2000_, among both men and women, there were no significant differences in OR by cancer type.

**TABLE 5:** Odds ratio (OR) estimates for opioid use for long‐term cancer survivors versus controls without a cancer diagnosis in Cohort 2

	All		Men		Women		*p*‐value interaction (sex by cancer status)
	Bas^a^	Adjusted^b^	Base^a^	Adjusted^b^	Base^a^	Adjusted^b^	Adjusted^b^
	OR (95% CI)						
Any opioid use	1.23 (1.15–1.31)	1.23 (1.16–1.32)	1.23 (1.11–1.34)	1.22 (1.12–1.33)	1.24 (1.12–1.38)	1.25 (1.13–1.39)	0.75
Chronic use^c^	0.93 (0.81–1.08)	1.05 (0.90–1.21)	1.09 (0.88–1.35)	1.09 (0.88–1.36)	1.01 (0.82–1.23)	1.02 (0.83–1.25)	0.77
High use^d^	1.22 (0.77–1.91)	1.32 (0.84–2.07)	1.06 (0.51–2.2)	1.03 (0.50–2.1)	1.62 (0.9–2.9)	1.61 (0.91–2.85)	0.23
MME_2000_ ^e^	1.16 (1.05–1.27)	1.17 (1.06–1.29)	1.21 (1.06–1.39)	1.22 (1.06–1.39)	1.11 (0.97–1.27)	1.13 (0.99–1.29)	0.10

Note: ORs are for use from 2011 to 2015.

Referent category is individuals without a cancer diagnosis during the study window.

Abbreviation: MME, morphine milligram equivalent.

^a^ Model adjusted for sex (in non‐sex‐specific analyses) and age.

^b^ Model additionally adjusted for education, race/ethnicity, income, and marital status.

^c^ Chronic opioid use defined 90 consecutive days of opioid use allowing for 7‐day gap between refills.

^d^ High use defined as greater than or equal to 120 mg or any 90‐day period and at least 90 days of opioid use.

^e^ Total MME dose >2,000 mg.

## 4 DISCUSSION

We assessed opioid use among patients diagnosed in the United States with multiple cancer types at two points on the cancer care continuum––long‐term survivors and those within 2 years of cancer diagnosis (short‐term). Any opioid use was similar among long‐term cancer survivors compared to controls, but differed among short‐term survivors for any opioid use and marginally for chronic opioid use. We observed differences in opioid prescription fills by cancer status and among the two cohorts. Compared to controls, short‐term survivors had higher rates of any opioid use and chronic use. In the long‐term cohort, any use was similar. Though rare in both cohorts, high use was significantly more frequent among short‐term survivors than controls.

These observations may indicate pain prevalence among cancer patients despite increased awareness of the importance of pain management.^1^, ^32^ High use among the short‐term cohort is expected and may be explained by proximity of treatment. Specifically, patients in this group may experience pain requiring pain management as a result of surgery, chemotherapy, or radiation. Concerns of inadequate pain relief among cancer patients may not be sufficiently assessed in this study. The window ends in the year before the CDC released opioid management guidelines in 2016. Though the guidelines were explicitly not intended for cancer patients or individuals who receive palliative care, these patients have been affected by guideline adoption.^7^ This may explain why any opioid use rates among cancer patients in this study population were higher than non‐cancer patients.^8^ Future studies should assess change in opioid prescribing rates among older cancer patients after release of the CDC guidelines.

Opioid use patterns at end‐of‐life likely vary. Though we do not have information on recurrence or progression after diagnosis, we assessed the effect of mortality from cancer on opioid use. In the long‐term cohort, 5.1% of patients died from cancer during or within a year of the end of the assessment period. In a sensitivity analysis excluding these patients, chronic use decreased from 5.2% to 4.9% and MME_2000_ decreased from 13.6% to 12.2%. To be included in the short‐term cohort, participants had to survive to 2013 to consent to linkage. Therefore, the cancer death rate may be lower than otherwise expected; within 3 years of diagnosis 12.0% had a cancer‐specific death.

Little increased rates of chronic use for short‐term survivors compared to controls may explain why long‐term survivors had little increase of any opioid use and no increase in chronic use compared to controls. This may indicate cancer patients do not become addicted after cancer diagnosis and initial treatment or reflect a shift to use of non‐opioids for pain management. Near constant opioid use rates over time was observed in a study of Canadian cancer patients 65 years and older.^21^ In a SEER‐Medicare population, cancer patients 6 years post‐diagnosis were no longer more likely to be chronic users when compared to controls.^30^ In contrast, a study of Medicare beneficiaries in Texas diagnosed between 1995 and 2008 reported prolonged high opioid use rates at 5 years after cancer diagnosis and longer.^13^ Variability in opioid rates by location, sex, and other factors has been documented.^33^ Chronic opioid use rates over time may differ among younger (<64 years) cancer patients.^34^

Much of the increase in opioid use in the short‐term cohort was observed within the first year after diagnosis which may be due to receipt of treatment. Site‐specific treatments such as surgery, chemotherapy, or radiation, symptoms or disease progression, and cancer stage likely contribute to opioid use patterns, thus, the observed higher rates of chronic use and any opioid use among cancer patients when compared to controls is expected. We included multiple cancer types and patients likely received varying treatments specific to their diagnosis. Approximately 10% of patients in the short‐term cohort had metastatic (stage IV) disease at diagnosis while 3% of patients in the long‐term cohort had metastatic disease at diagnosis. Differences in treatment by cancer site may explain the differences observed in opioid use by sex. The ORs for any opioid use comparing short‐term survivors to controls were 4.1 versus 2.8 for women and men, respectively (*p* for interaction = 0.002). Among women with breast cancer (about 17% of the short‐term cohort and 19% of the long‐term cohort), most received surgery, which may result in postoperative pain requiring pain management via opioids, while for prostate cancer (nearly 22% of the short‐term cohort and 41% of the long‐term cohort), about 50% and 33% received surgery for long‐term and short‐term cancer survivors, respectively.

This study comprised a population enrolled in Medicare Part D and may not represent individuals enrolled in other prescription drug plans, who may have differing patterns of opioid use.^35^ Opioid use represents prescriptions filled under Part D and paid for by Medicare. Information on medication use was not available. We do not know if medication was used as prescribed. Indications were not available and we were unable to determine if medications were prescribed for cancer‐related pain, the appropriateness of medication use, or if medication use improved pain and other cancer‐related symptoms. We were unable to ascertain PRN use of opioids. While non‐cancer disease occurring after baseline was captured using the supplemental questionnaire (2006–2007), later occurrence of disease that may have required prescriptions for pain management was not captured.

Differences in opioid use over time and within subgroups of cancer patients highlight the importance of monitoring opioid use during a patient's cancer trajectory possibly used in combination with other strategies for pain management and reduction of misuse.^20^, ^36^ Research is needed within subgroups of cancer patients to differentiate overuse from appropriate use based on treatment and survivorship plans and patient goals. This may include assessing chronic high or low use of specific opioids or describing other medications commonly used with opioids.^37^

Though we observed no difference between short‐ or long‐term cancer patients and controls with regard to the number of comorbidities, they may influence opioid use. Future research should examine concurrent use of medications commonly used to manage comorbidities. Opioid use in the 6–18‐month period prior to diagnosis or pseudo‐diagnosis in the short‐term cohort was similar in cases and controls, suggesting the observed higher rate of opioid use in cases post‐diagnosis was not due to confounding based on higher rates in cases of non‐cancer conditions predisposing to opioid use. Finally, the current study included PLCO trial volunteers who may be healthier than the general U.S. population. The impact of a healthy volunteer effect on outcomes in this trial has been reported elsewhere^38^ and could explain why opioid use rates were mainly similar. In a sample of community participants, it may be important to consider how other patient‐level factors impact opioid use among cancer survivors.

## 5 CONCLUSION

Any opioid use was similar among long‐term cancer survivors compared to controls, but differed among short‐term survivors for any opioid use and marginally for chronic opioid use. Future research should consider concomitant use of opioids and non‐opioid medications commonly used to manage pain among cancer patients.

## ACKNOWLEDGMENTS

The authors wish to thank the Prostate, Lung, Colorectal, and Ovarian (PLCO) screening trial participants. More information about PLCO and requests for access to the publicly available data can be found at https://cdas.cancer.gov/plco/.

## CONFLICTS OF INTEREST

The authors have no conflicts of interest to disclose.

## AUTHOR CONTRIBUTIONS

DD, SS, and PP contributed to the design and analysis of the research. PP secured access to the data. DD, SS, and PP drafted and significantly reviewed and revised the manuscript.

## Data Availability

More information about the Prostate, Lung, Colorectal, and Ovarian Cancer Screening Trial (PLCO) and requests for access to the publicly available data that supports this work can be found at https://cdas.cancer.gov/plco/.

## Appendix

**FIGURE A1** Participants included in the study sample by cancer status and time since cancer diagnosis

**TABLE A1** Frequency of cancer type by cohort and sex

**Table:**

	Short‐Term (Cohort 1)			Long‐Term (Cohort 2)		
	All	Men	Women	All	Men	Women
Cancer type	N (%)	N (%)	N (%)	N (%)	N (%)	N (%)
Bladder	46 (3.1)	36 (4.5)	10	147 (3.4)	113 (4.2)	34 (2.0)
Breast	252 (16.9)	2 (0.3)	250 (36.3)	824 (18.8)	6 (0.2)	818 (47.8)
Colorectal	112 (7.5)	51 (6.4)	61 (8.9)	336 (7.7)	179 (6.7)	157 (9.2)
Endometrial	48 (3.2)	‐	48 (7.0)	165 (3.8)	‐	165 (9.7)
Kidney	52 (3.5)	36 (4.5)	16 (2.3)	102 (2.3)	61 (2.3)	41 (2.4)
Leukemia/Lymphoma	152 (10.2)	81 (10.1)	71 (10.3)	252 (5.8)	125 (4.7)	127 (7.4)
Lung	124 (8.3)	59 (7.4)	65 (9.4)	137 (3.1)	58 (2.2)	79 (4.6)
Melanoma	110 (7.4)	62 (7.7)	48 (7.0)	231 (5.3)	148 (5.6)	83 (4.9)
Oral Cavity	43 (2.9)	29 (3.6)	14 (2.0)	53 (1.2)	31 (1.2)	22 (1.3)
Ovarian	15 (1.0)	‐	15 (2.2)	38 (0.9)	‐	38 (2.2)
Pancreatic	19 (1.3)	9 (1.2)	10 (1.5)	5 (0.1)	2 (0.1)	3 (0.2)
Prostate	320 (21.5)	320 (39.9)	‐	1,809 (41.3)	1,809 (67.9)	‐
Thyroid	18 (1.2)	8 (1.0)	10 (1.5)	62 (1.4)	17 (0.6)	45 (2.6)
Other/unknown/Ill‐defined	180 (12.1)	109 (12.5)	71 (10.3)	216 (4.9)	117 (4.4)	99 (5.6)

**TABLE A2** Comparison of the demographic distribution among study eligible and those excluded from current study, Cohort 1

**Table:**

		Short‐term cancer survivors N = 1,491	Ineligible N = 3,491	All assessed for eligibilityN = 4,982
Age at start of analysis period	Median (IQR)	75 (71/80)	76 (71/80)	76 (71/80)
		N (col %)	N (col %)	N (col %)
Sex	Men	802 (53.8)	109 (54.7)	2,711(54.4)
	Women	689 (46.2)	802 (53.8)	2,271 (45.6)
Education	Less Than High School	48 (3.2)	238 (6.8)	286 (5.7)
	High School Graduate	258 (17.3)	756 (21.7)	1,014 (20.4)
	Post High School	497 (33.3)	1,113 (31.9)	1,610(32.3)
	College Graduate	665 (44.6)	1,267 (36.3)	1,932 (38.8)
	Unknown	23 (1.5)	117 (3.4)	140 (2.8)
Race/Ethnicity	White, Non‐Hispanic	1,353 (90.7)	2,992 (85.7)	4,345 (87.2)
	Black, Non‐Hispanic	28 (1.9)	204 (5.8)	232 (4.7)
	Hispanic	17 (1.1)	47 (1.4)	64 (1.3)
	Asian	60 (4.0)	116 (3.3)	176 (3.5)
	Pacific Islander/American Indian	12 (0.8)	18 (0.5)	30 (0.6)
	Unknown	21 (1.4)	114 (3.3)	135 (2.7)
Marital Status	Married/Living as Married	1,103 (74.0)	2,533 (72.6)	3,636 (73.0)
	Widowed	172 (11.5)	423 (12.1)	595 (11.9)
	Divorced/Separated	151 (10.1)	366 (10.5)	517 (10.4)
	Never Married	68 (4.6)	114 (3.3)	174 (3.5)
	Unknown	5 (0.3)	55 (1.6)	60 (1.2)

*Note:* To be potentially eligible for study inclusion, the participant had to have a cancer diagnosis during the period 2011–2013 and had to survive at least a year from cancer diagnosis to have 1 year of medication use data.

**TABLE A3** Comparison of the demographic distribution among study eligible and those excluded from current study, Cohort 2

**Table:**

		Long‐term cancer survivors N = 4,377	Ineligible N = 12,818	All assessed for eligibility N = 17,195
Age at Start of Analysis Period	Median (IQR)	75 (71/79)	76 (72/81)	76 (71/80)
		N (col %)	N (col %)	N (col %)
Sex	Men	2,666 (60.9)	7,347 (57.3)	10,013 (58.2)
	Women	1,711 (39.1)	5,471 (42.7)	7,182 (41.8)
Education	Less Than High School	147 (3.4)	868 (6.8)	1,013 (3.4)
	High School Graduate	752 (17.2)	2,836 (22.1)	3,588 (20.9)
	Post High School	1,372 (31.4)	4,113 (32.0)	5,485 (31.9)
	College Graduate	2,041 (46.6)	4,560 (35.6)	6,601 (38.4)
	Unknown	65 (1.5)	441 (3.4)	506 (2.9)
Race/ethnicity	White, Non‐Hispanic	3,946 (90.2)	11,102 (86.6)	15,048 (87.5)
	Black, Non‐Hispanic	114 (2.6)	645 (50)	759 (4.4)
	Hispanic	52 (1.2)	206 (1.6)	258 (1.5)
	Asian	177 (4.0)	375 (2.9)	552 (3.2)
	Pacific Islander/American Indian	32 (0.7)	82 (0.6)	114 (0.7)
	Unknown	56 (1.3)	408 (3.2)	464 (2.7)
Marital status	Married/Living as Married	3,377 (77.2)	9,322 (72.7)	12,699 (73.9)
	Widowed	495 (11.3)	1,617 (12.6)	2,112 (12.3)
	Divorced/Separated	370 (8.5)	1,257 (9.8)	1,627 (9.5)
	Never Married	119 (2.7)	398 (3.1)	517 (3.0)
	Unknown	16 (0.4)	224 (1.8)	240 (1.4)

*Note:* To be potentially eligible for study inclusion, the participant had to have a cancer diagnosis before the end of 2008 and had to survive at least 1 year from 2011 on to have a year of medication use data.

**TABLE A4** Most common opioid medications prescribed by proportion of morphine milligram equivalents (MME) and proportion of prescriptions among cancer patients and controls, Short‐term survivors and controls, Cohort 1

**Table:**

Medication	% of MME dose		% of prescriptions	
	Controls	Cases	Controls	Cases
Tramadol HCL	19.4	9.8	23.7	14.8
Hydrocodone/Acetaminophen	20.5	16.8	30.8	28.2
Fentanyl	13.0	14.1	2.5	4.9
Hydrocodone Bitartrate/Acetaminophen	9.5	7.1	14.0	16.1
Oxycodone HCL	11.9	20.3	6.6	10.5
Oxycodone HCL Acetaminophen	9.1	12.1	9.7	13.6
Morphine Sulfate	7.4	14.3	2.0	3.9
Acetaminophen w/codeine	3.4	1.8	7.1	5.3

*Note:* Includes all medications with at least 5% of MME dose or prescriptions in either cases or controls.

**TABLE A5** Most common opioid medications prescribed by proportion of morphine milligram equivalents (MME) and proportion of prescriptions among cancer patients and controls, Long‐term survivors and controls, Cohort 2

**Table:**

Medication	% of MME dose		% of prescriptions	
	Controls	Cases	Controls	Cases
Tramadol HCL	19.1	17.4	23.7	20.3
Hydrocodone/Acetaminophen	18.8	21.6	27.7	29.6
Fentanyl	13.7	9.2	2.6	2.2
Hydrocodone Bitartrate/Acetaminophen	10.4	10.7	16.2	16.3
Oxycodone HCL	12.4	11.8	6.9	7.4
Oxycodone HCL/Acetaminophen	9.7	12.2	10.1	11.9
Morphine Sulfate	6.8	7.2	2.0	2.0
Acetaminophen with codeine	3.4	2.7	7.2	6.4

*Note:* Includes all medications with at least 5% of MME dose or prescriptions in either cases or controls.
